# In-situ tool wear monitoring and its effects on the performance of porcine cortical bone drilling: a comparative in-vitro investigation

**DOI:** 10.1186/s40759-017-0019-z

**Published:** 2017-01-25

**Authors:** Vishal Gupta, Pulak M. Pandey

**Affiliations:** Mechanical Engineering Department, Indian Institute of Technology, Delhi, New Delhi 110016 India

**Keywords:** Drill bit, Hollow drill, Drilling, Diamond Grain, Tool Wear, Bone

## Abstract

**Background:**

Drilling is one of the most widely used process in orthopaedic surgical operation and the same drill bit is used a number of times in hospitals. Using the same drill bit a several times may be the cause of osteosynthesis and osteonecrosis.

**Methods:**

In the present work, the effect of repeated orthopaedic surgical twist drill bit on the tool wear, force, torque, temperature and chip morphology during porcine cortical bone drilling is studied. Results were compared with rotary ultrasonic drilling (RUD) on the same bone using a hollow drill tool coated with diamond grains. A sequence of 200 experiments (100 with each process, RUD and CD) were performed with constant process parameters.

**Results:**

Wear area on the drill bit is significantly increased as the drill bit is used repeatedly in CD, whereas no attritious wear was found on the diamond coated grains in RUD.

**Conclusions:**

Comparative results showed that cutting force, torque and temperature increased as a function of tool wear in CD as the same drill bit was used a number of times. No significant variation in the cutting force and torque was observed in RUD as the number of drilled holes increased.

## Background

In orthopedic or trauma surgical operations drill bit is used number of times to make holes in bones. Using the same twist drill bit a number of times leads to decrease in its cutting efficiency during the bone drilling process. A significant tool wear is formed on the cutting edges of the drill bit due to reuse, which may lead to increase in the frictional forces and heat between the bone and the drill bit. In this direction, few studies have been reported in the medical field, which are summarized below:

(Allan et al. 2005) performed in vitro study on a pig mandible to investigate the effects of drill wear on the change in bone cutting temperature using 3 different drill bits (*n =* 0, *n =*600 holes drilled, *n =* used many times in operation theater). They reported that with increase in the drill wear, temperature increased significantly. It was also observed that the drill bit obtained from the operation theater generated a maximum rise in temperature as compared to the other two types of drill bits.

(Oliveira et al. 2012) conducted drilling experiments on bovine bone with twisted stainless steel and ceramic drills to find out the relation between the thermal changes and drill wear. They found that temperature increased with the drilling depth and thickness of cortical bone. Their investigations suggested that depth was the predominant factor influencing the temperature variation in bone drilling. Further, it was concluded that stainless steel drill bit had more tip wear as compared to the ceramic drill bit.

(Chacon et al. 2006) investigated the effect of repeated drill bit (25 times) on temperature using a femura cortical bone and found that temperature significantly increased with the use of repeated drill bit. They reported that drill tool was worn after being used a number of times.

(Queiroz et al. 2008) performed the study on a rabbit’s tibiae to investigate the effect of repeated drill bit (40 times) on the bone cell viability. They concluded that cell viability decreased in the bone matrix using a repeated drill bit. SEM was used to analyze the wear on the drill bit and they observed that it increased consistently. In another in-vitro study on rabbits, (de Souza et al. 2011) concluded that significant heat was generated if the same drill bit was reused after 50 times and a worn drill bit could damage the bone tissue during drilling.

(Jochum and Reichart 2000) investigated the influence of titanium repeated drill bit, of diameter 3.2 mm (51 times) on the temperature using pig mandibles. In their in vitro study, rotational speed was 1200 without any irrigation. They reported a significant amount of increase in temperature if the same drill bit was used more than 40 times. They also found that the sharpness of the drill bit edge weakened, if used number of times.

Recently (Staroveski et al. 2015) performed an experimental investigation on cortical bone to study the effect of tool wear on force and torque. They reported that temperature continuously increased using a repeated uses of drill bit.

In the last three decades, various studies have been reported on the monitoring of drill wear which is focused on industrial applications (Jantunen 2002). However, drilling method in surgical operation theater is different as compared to industrial application. In trauma or orthopaedic surgical drilling, temperature should be lower than 47 °C to avoid thermal necrosis (Lundskog 1971; Eriksson et al. 1984; Krause 1987; Augustin et al. 2008). Cutting forces (Alam et al. 2009; Alam et al. 2011; Wang et al. 2014a) and torque (Alam et al. 2011; Wang et al. 2014a) should also be minimum. Using a drill bit repeatedly increased the temperature (Jochum and Reichart 2000; Allan et al. 2005; Chacon et al. 2006; Queiroz et al. 2008; de Souza et al. 2011; Oliveira et al. 2012) and force (Staroveski et al. 2015) significantly which may cause to decrease the strength of internal fixation (Allan et al. 2005).

Measurement of drill tool wear during bone drilling is not possible, but it can be measured by the wear area on the tool after the drilling. In the present study, tool wear on drill bit during the CD of porcine cortical bone has been measured using the white light microscope and its effect on the cutting force, torque and temperature has also been studied. Results were compared with the RUD using a hollow drill tool coated with diamond grain particles. The aim of the present study is to introduce the RUD on bone and to investigate the effect of tool wear on the cutting performance.

## Methods

### Bone specimen details

In-vitro comparative study has been performed on the male diaphyseal part of porcine cortical bone (thickness of 4–5 mm) which was obtained immediately after slaughtering. Four porcine cortical bones were purchased from the local slaughterhouse and preserved immediately in a buffered solution of 10% formalin and 90% saline water. The porcine was around one year old and 88 kg in weight. The proximal and distal ends of the cortical bone have been removed by using a plane hacksaw.

### In vitro experimentations

**Fig. 1:**
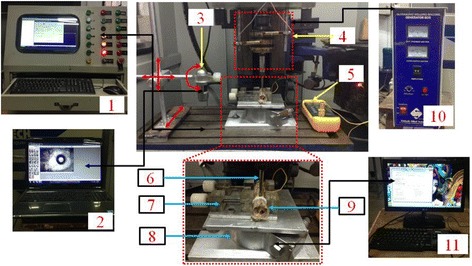
Experimental setup and equipment’s used for force, torque, temperature and tool wear measurement. 1) CNC controller unit, 2) personal computer attached to microscope, 3) Dino light microscope, 4) RUD tool assembly, 5) Digital thermometer with thermocouple, 6) Tool, 7) Bone holding fixture, 8) Bone, 9) 6- axis dynamometer, 10) Ultrasonic generator unit and 11) personal computer attached to dynamometer

**Fig. 2:**
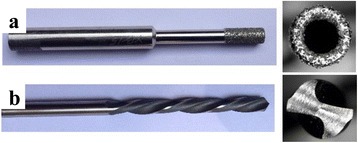
Actual images of drill tool used in the present study (**a**) designed hollow tool and (**b**) twist drill bit

In vitro experiments were performed on the x-y-z axis of the CNC milling machine. CD without any ultrasonic vibrations was performed by using a new orthopedic drill bit. Existing machine was modified to perform the RUD with designed hollow tool as shown in Fig. 1. Figure 2 shows the actual image of the designed hollow tool and orthopaedic twist drill bit. Specification of the hollow drill tool and twist drill bit has been listed in Table 1. Designed RUD setup was working with vibrational frequency and amplitude of 20 kHz and 4–20 μm respectively. Ultrasonic vibrations were given to the hollow drill tool through the ultrasonic power generator. Vibrations were transmitted to the tool by the booster/horn. Bone sample was placed perpendicular to the tool for performing both types the drilling. As the bones were unsymmetrical in nature, special bone holding fixture was designed and fabricated for holding the bone sample for safe drilling.

**Table 1:** Specification of hollow drill tool and twist drill bit

Diamond coated hollow drill tool		Twist drill bit	
Outer diameter	4.5	Number of cutting lips	2
Inner diameter	2.9	Helix angle:	8.4°
Mesh size	80–100	Point angle	120°
Areal average abrasive/grain density	15 abrasive/mm^2^	Tool base material:	Stainless steel
Abrasive/grain type	Diamond		
Tool shank material	EN-31		
Abrasive/grain coated method	Electroplating		

A stand with adjustment features was designed and fabricated for holding the microscope (in situ tool wear measurement). Horizontal and vertical axis (X-Y-axis) was adjustable with sliding movements and Z- axis was the base of the stand which was also movable. A rotational feature was provided for rotational movement (0° to 360°) of the microscope with horizontal arm (X-axis) as shown in Fig. 1.

A sequence of 200 experiments were performed at room temperature, (100 with each drilling process) without any irrigation, using constant process parameters i.e., spindle speed of 1500 rpm, feed rate of 10 mm/min, and drill diameter of 4.5 mm. Vibrational frequency of 20 kHz and amplitude of 16 μm was used for the RUD. These parameters were selected on the basis of the in vitro bone drilling study performed by (Gupta and Pandey 2016a; Gupta and Pandey 2016b). Drilled holes were made to a depth of 4 mm with both the processes.

### Data acquisition

Dino Lite microscope (Dino-Lite Pro II AM411T) was used to capture the images of the wear on the drill tools used in this work. Images were captured at *n =* 0, i.e. new drill tool and thereafter for every 5th experiment (5th, 10th, 15th….100th). These images have been further analyzed in the microscopic image analyzer software (Digimizer) for measurement of the wear on the respective tools. The Chip morphology was also studied after the 1st, 5th, 10th, 15th …. 100th experiments. For measurement of wear on the drill bit, 21 images were captured for CD and 63 for hollow drill tool, and for chip morphology a total of 42 images were captured for both the drill tools.

In total 126 values were measured (63 with each process) for cutting force (42), torque (42) and temperature (42). 6 axis Schunk dynamometer (Delta IP68) was used to measure the cutting forces and torque, which was mounted on the table of the machine (Fig. 1). Temperature was measured by digital thermometer with thermocouple (K-type, Cu-Al) probe. The probe of the thermocouple was inserted (in a predrilled hole of diameter 0.6 mm) to a depth of 4 mm at a distance of 0.5 mm from the drill test hole. Cutting force, torque and temperature were measured simultaneously at the 1st, 5th, 10th, 15th …. 100th experiments for CD and RUD. All the mentioned measurement equipment’s were calibrated and checked before performing the actual experiments.

## Results

### Tool wear mechanism

**Fig. 3:**
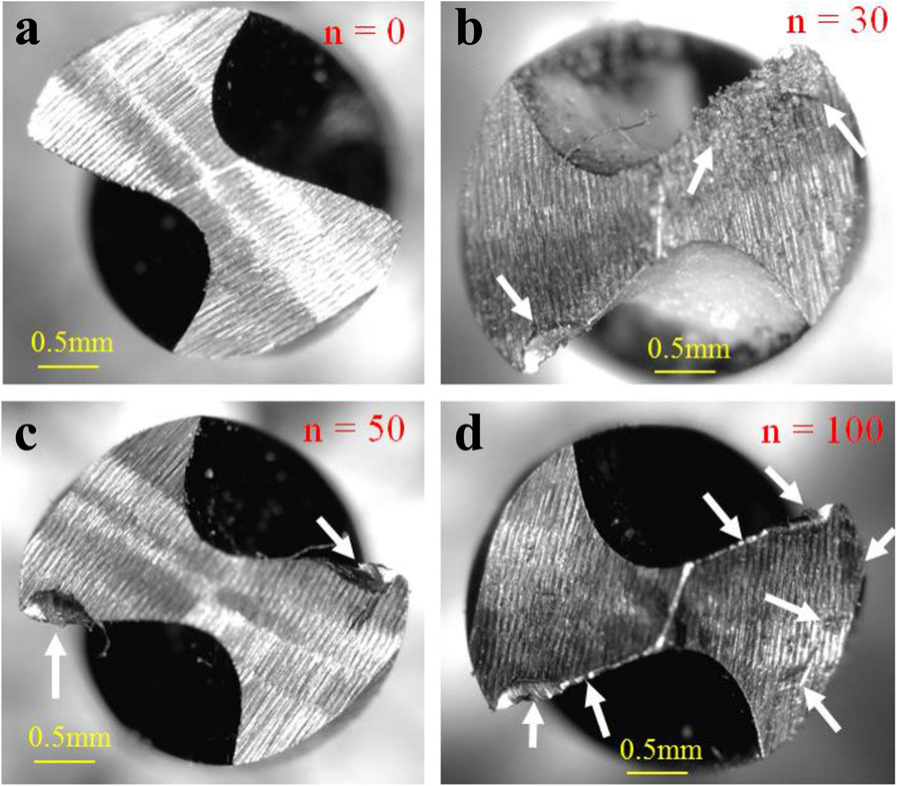
Microscopic images of wear on conventional drill bit using a Dino lite microscope (**a**) new drill bit (**b**) after 30 drills (**c**) after 50 drills (**d**) after 100 drills

**Fig. 4:**
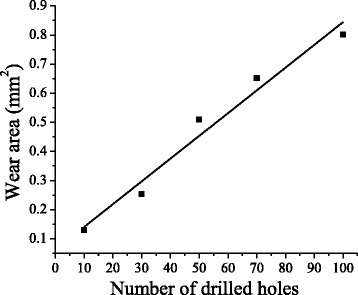
Effect of repeated drill tool on the wear area in CD

Figure 3 shows typical wear on the conventional orthopaedic drill bit after making 30, 50 and 100 holes. It can be noted from Fig. 3a that there is no wear on the new drill bit i.e. before start of experimentation. Tool wear occurred at the main cutting edge of drill bit as the repeated drill bit was used number of times [Fig. 3(b-d)]. Tool wear area on the cutting edge of flank was measured using a Dizimizer software. Figure 4 shows that tool wear area increased linearly with the increase in number of drilled holes.

**Fig. 5:**
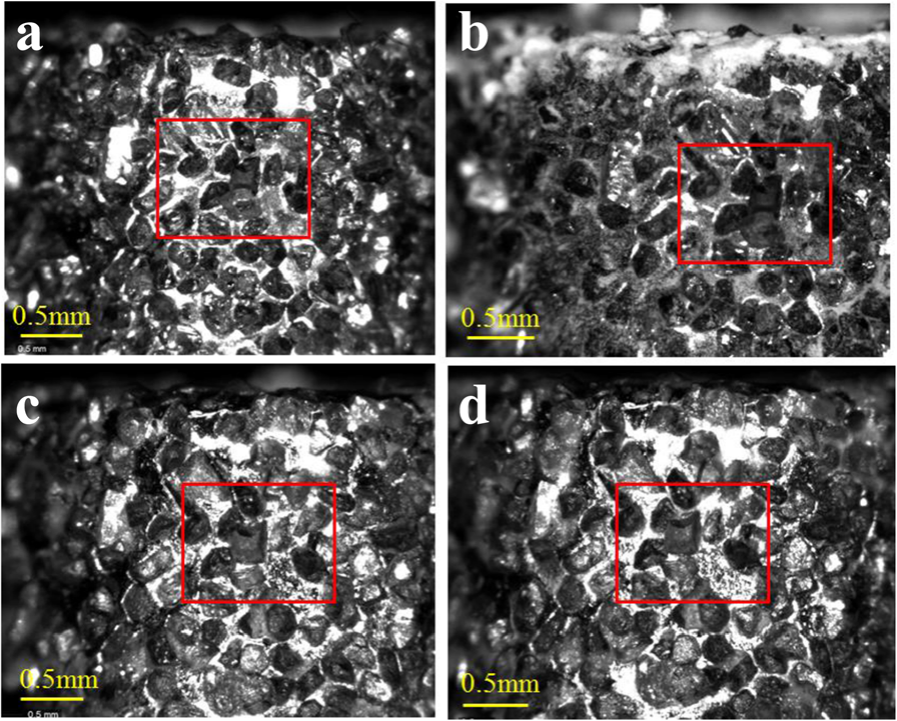
Microscopic images of hollow drill tool using a Dino lite microscope (**a**) new drill bit (**b**) after 30 drills (**c**) after 50 drills (**d**) after 100 drills

Topography of the diamond coated hollow drill tool was also observed before experiments (*n =* 0) and after drilled holes (5th, 10th …… 100th) at three different locations on the periphery of the hollow drill tool. The same area of the hollow drill tool was investigated every time. Figure 5(a) shows topography of the hollow drill tool at one particular location before performing experiments and after making 30, 50 and 100 holes [Fig. 5(b-d)] respectively. After performing the 100th test using the same hollow drill, no attritious wear was found on the diamond grains [Fig. 5(d)]. Similar observations were made at two other locations on the hollow drill tool.

### Cutting force and torque

**Fig. 6:**
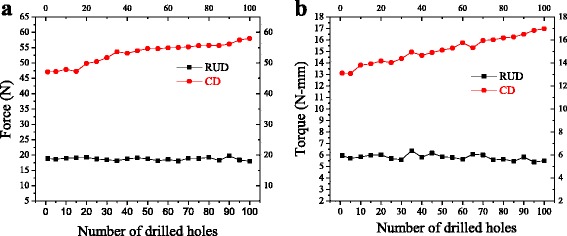
Effect of reused drill tool on maximum cutting (**a**) force and (**b**) torque, for both the drilling techniques

The change in the magnitude of maximum cutting force and torque with respect to repeated drilling for CD (without any ultrasonic vibration) and RUD are presented in Fig. 6. It was observed that in CD, maximum cutting force [Fig. 6(a)] and torque [Fig. 6(b)] increased significantly as the drill bit was reused. An increase in the cutting force and torque was observed to be 18.8% and 22.8% respectively, when the number of drilled hole increased from 1st to 100th experiment. The increase in the cutting force and torque with repeated drilling is due to the increase in the worn area of the tool. Similar results were obtained for the cutting force (Park et al. 2011; Wang et al. 2014b; Çelik et al. 2015) and torque (Park et al. 2011; Wang et al. 2014b) during drilling of carbon fiber reinforced polymer, but the magnitude of cutting force and torque was different.

On the other hand, no significant effect on cutting force and torque was observed while using a reused hollow drill tool in RUD on cortical bone [Fig. 6]. Whereas, (Cadorin and Zitoune 2015) reported that cutting force increased to 16% after the 100th drilled hole w.r.t. 1st drilled hole while drilling on a carbon fiber reinforced polymer with hollow drill tool coated with diamond particle (without any ultrasonic vibration).

### Temperature and chip morphology

**Fig. 7:**
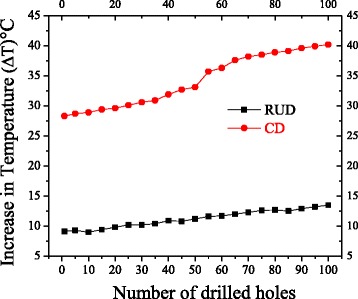
Effect of repeated drill tool on maximum temperature, for both the drilling techniques

The change in the temperature of cortical bone using repeated drilling for both the drilling processes is summarized in Fig. 7. It can be seen that temperature increased significantly for both the drilling process as the number of drilled holes increased from 1st to 100th experiment. The change in the value of temperature was 28.3 °C and 40.2 °C for the 1st and 100th drilled hole respectively in CD and 9.1 °C and 13.5 °C in RUD. With respect to RUD, the change in temperature as compared to CD dropped by 67.8% for 1st drilled hole and 66.4% for 100th drilled hole.

**Fig. 8:**
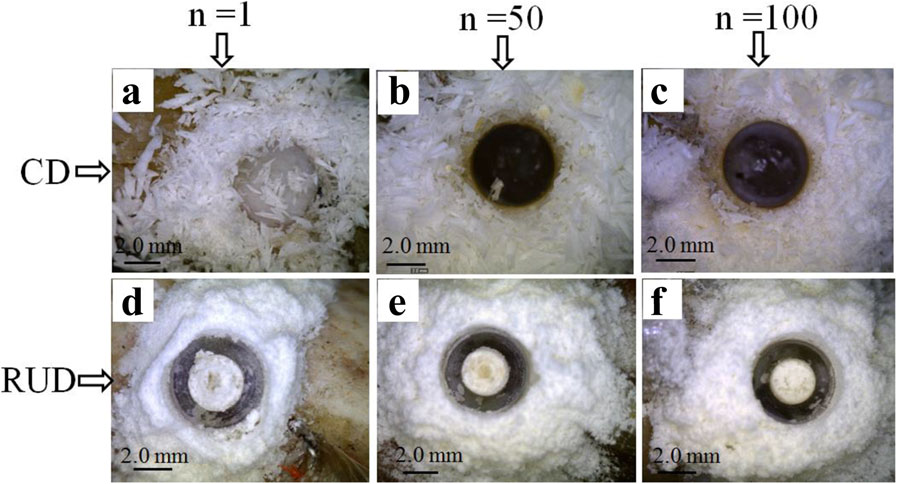
Effect of tool wear on chip morphology for both the drilling technique

Microscopic images were captured to observe the change in chip morphology with repeated drilling for both the processes as shown in Fig. 8. In CD, long chips were formed in the 1st experiment [Fig. 8(a)] fragmented chips were observed in the 50th experiment [Fig. 8(b)] and a further reuse of the same drill bit resulted in powdery and fragmented chips in the 100th experiment [Fig. 8(c)]. It may be due to that the cutting edge becomes dull after using a same drill bit for number of times. While RUD generated powder form of chips, and no change in the chip morphology was observed [Fig. 8(e-f)]. Increase in the number of drilled holes and change in the color of chips was also observed in CD. This was due to the use of same drill bit, the temperature continuously increased, as discussed. However no change in the color of chips was observed in RUD, as this process generated much lower cutting temperature as compared to CD.

### Statistical analysis

Analysis of variance (ANOVA) was used to perform the statistical analysis using the OriginPro 8 software. Linear fit model was used to predict the significant difference between the two drilling processes, i.e., *p < 0.05*, on the force, torque and temperature. Table 2 represents the ANOVA of force, torque and change in temperature for RUD and CD.

**Table 2:** ANOVA of force, torque and temperature for RUD and CD

			DF	SS	MS	F	*P*	R^2^ Adj.	Remarks
Force	RUD	Model	1	0.12898	0.12898	0.62275	0.43976	–0.019	*p > 0.05* Insignificant
		Error	19	3.93501	0.20711				
		Total	20	4.06398					
	CD	Model	1	26.03881	218.52432	178.24016	4.1915E-11	0.898	*P < 0.05* Significant
		Error	19	0.8901	0.04685				
		Total	20	26.92891					
Torque	RUD	Model	1	0.05072	0.05072	0.40808	0.53057	–0.030	*p > 0.05* Insignificant
		Error	19	2.3614	0.12428				
		Total	20	2.41211					
	CD	Model	1	26.03881	26.03881	555.81918	1.5543E-15	0.965	*P < 0.05* Significant
		Error	19	0.8901	0.04685				
		Total	20	26.92891					
Temperature	RUD	Model	1	40.70315	40.70315	1318.87515	0	0.985	*P < 0.05* Significant
		Error	19	0.58638	0.03086				
		Total	20	41.28952					
	CD	Model	1	369.77029	369.77029	522.3633	2.77556E-15	0.963	*P < 0.05* Significant
		Error	19	13.44971	0.70788				
		Total	20	383.22					

Statistically significant difference was found from the ANOVA analysis (Table 2) between the two drilling processes on the force and torque for repeated drilling. Insignificant effect on the force and torque was found for repeated hole drilling by RUD, as their *p* values are greater than 0.05, and adjusted R^2^ for force and torque is also–0.019 and –0.030 respectively. In other words, a statistically significant effect on force and torque was found for CD. *P* value of force and torque was found to be less than 0.05 and corresponding adjusted R^2^ is 0.898 and 0.965 respectively.

A positive correlation was found between the two drilling methods on the increase in temperature for the repeated drilled holes. RUD and CD produced a statistically significant effect on the rise in temperature (*P < 0.05*), and their adjusted R^2^ is 0.985 and 0.963 respectively.

## Discussions

The change in the cutting force, torque (Alam et al. 2011) and temperature (Alam and Silberschmidt 2014) is affected by the drilling parameters including the feed rate, applied force, spindle speed, drill geometry, cooling system etc. In this work, effect of repeated drilling by two drilling methods i.e. CD and RUD were compared for the cutting force, torque, temperature, tool wear and chip morphology. RUD is a non-traditional machining process in which ultrasonic vibrations are given to the hollow drill tool. Cutting mechanism in this process is different as CD (Gupta et al. 2016; Gupta and Pandey 2016a; Gupta and Pandey 2016b). In RUD the tool work contact ratio is minimal due to the ultrasonic vibrations, whereas in CD, there is direct contact between the tool and the workpiece. As a result, more frictional forces are generated in CD as compared to RUD. Bone is different than metal or composite as it is a complex material (Yu et al. 2005) in the form of layers containing a soft tissue, hard tissue, bone marrow and protein fibers. The phenomena of wear on the tool is characterized by friction between the drill bit and workpiece (Park et al. 2011; Çelik et al. 2015).

As the number of drilled holes increase, the cutting edge of a drill bit becomes dull and its sharpness diminishes which may be the cause of increase in the friction between the drill tool and bone in CD. The material is eroded from the tool in micro and sub - micro level [Fig. 3(b-d)]. Therefore, cutting forces, torque and temperature increase significantly as the number of drilled holes increase.

In RUD, material is removed by the cutting action of the diamond grain and hammering action of ultrasonic vibrations, so intermediate contact is generated between the workpiece and the bone. As a result, less amount of friction is generated. Therefore, no wear on the abrasive particles was observed on the hollow drill tool and no dislodgment of the diamond grains was also observed after the 100th experiment.

However, (Zeng et al. 2005) reported that after the 16th experiment diamond grains were dislodged, while drilling a SiC with rotary ultrasonic machining. They also concluded that the color of thee diamond grains changed, due to increase in the temperature. But no change in the color of diamond grains was observed, in the RUD of bone, which also shows that temperature of these diamond grains was low during RUD. This may be due to low friction and temperature generated at the interface of the bone and diamond grains.

## Conclusions

Effect of repeated holes drilling on the force, torque, temperature and chip morphology was carried out as the function of tool wear, in the drilling of cortical bone for the two drilling processes. The following conclusions were drawn on the basis of the present in—vitro study:

1. The worn area on the orthopaedic twist drill bit increased as it was used a number of times in CD, while in RUD, no attritious wear was found on the diamond grains
2. In CD, cutting force, torque and maximum change in temperature increased continuously as the number of drilled holes increased. However, in RUD only cutting temperature increased gradually with repeated drill tool.
3. After performing the 100th experiment in RUD, no significant variation in the cutting force and torque was observed.
4. Chip morphology changed as the drill bit was used repeatedly in CD, while no change in chip morphology was observed in RUD.
5. RUD generated lower cutting force, torque and temperature with respect to CD, which can eliminate the burring of soft tissue in orthopaedic bone drilling process.
6. Statistical results also showed that reused drill bit had a significant effect on the cutting force, torque and temperature in CD.
